# Using the Health Belief Model to Analyze Instagram Posts about Zika for Public Health Communications

**DOI:** 10.3201/eid2501.180824

**Published:** 2019-01

**Authors:** Jeanine P.D. Guidry, Kellie E. Carlyle, Jessica G. LaRose, Paul Perrin, Marcus Messner, Mark Ryan

**Affiliations:** Virginia Commonwealth University, Richmond, Virginia, USA

**Keywords:** infectious diseases, behavior, internet, Instagram, posts, Zika, Zika virus, viruses, #Zika, health belief model, public health communications, social media

## Abstract

We analyzed Instagram posts about Zika by using the Health Belief Model. We found a high presence of threat messages, yet little engagement with these posts. Public health professionals should focus on posting messages to increase self-efficacy and benefits of protective behavior, especially when a vaccine becomes available.

Many persons will not engage in health protective behaviors without first understanding that they are at risk for an adverse outcome. However, the concept of risk can be difficult for persons to grasp (*1*), especially with a health topic such as Zika, with which persons are not likely to have much experience before an outbreak. Social media provides information that can help frame the public’s understanding of complex, highly contagious viruses (*2*). Instagram, especially, has potential for communicating risk information because visuals can increase attention and recall above those for text alone (*3*). In addition, women of reproductive age are particularly likely to use Instagram (*4*), making it a salient platform for Zika information. Analyzing Instagram posts about Zika lends insight into public attitudes and beliefs about Zika and the types of messages that are engaging. Understanding the target audience is a key step in the formative research process when designing effective prevention messaging.

Two studies have examined Zika-focused messages on Instagram (*5*,*6*), but neither study included health behavior theory, a major component of effective public health messaging (*7*). The Health Belief Model (HBM) provides a theoretical framework to explain the uptake of preventive behaviors by perceived susceptibility, severity, benefits, barriers, self-efficacy, and cues to action (*8*); HBM has been used successfully to develop health education messages and campaigns. To address the lack of health behavior theory in previous studies of Zika on social media, we examined the content of, and engagement with, Zika posts on Instagram through the lens of the HBM (Appendix).

We collected Instagram posts that used the hashtags #Zika and #ZikaVirus during August 1–31, 2016; hashtags, which are words/phrases preceded by the # symbol, create searchable topics on many social media platforms, including Instagram. We also used the simple random sampling in the social media mining tool Netlytic (https://netlytic.org) to select 1,000 posts for quantitative content analysis (intercoder reliability 0.71–1.00).

We found that of all HBM constructs, perceived severity (75.8%) and perceived susceptibility (59.9%) occurred most frequently, indicating that posts reflect a high level of perceived threat. However, posts mentioning fear and danger produced lower engagement (Table). One explanation for this finding might relate to how persons process a threat response. The Extended Parallel Processing Model provides 2 pathways as a threat response: danger control and fear control (*9*). Fear control takes place when a perceived threat is greater than the perceived efficacy to deal with the threat (e.g., a vaccine); as a result, responses are likely to be maladaptive. It remains to be determined whether this pattern holds once a high-efficacy response like the Zika vaccine becomes available, or whether engagement increases, as would be predicted by the danger control path of the Extended Parallel Processing Model.

**Table:** Mann-Whitney U test results for dichotomous independent variables and median engagement for the Health Belief Model in analyzing Instagram posts about Zika for public health communications

Engagement variable	Variable	Median present	Median absent	U*	Z	p value
Likes	Conspiracy theories	188.00	64.00	52,426.000	3.525	<0.001
Comments	Conspiracy theories	19.00	6.00	56,533.500	5.070	<0.001
Likes	Fear	61.00	102.00	78,434.000	−4.045	<0.001
Comments	Fear	6.00	10.00	78,619.000	−4.008	<0.001
Likes	Mosquito visual	57.50	77.50	63,749.500	−2.343	0.019
Likes	Person visual	89.50	57.50	141,628.000	3.733	<0.001
Likes	Perceived benefits prevention	45.00	78.50	46,042.000	−2.277	0.023
Comments	Perceived benefits prevention	4.00	7.00	45,176.000	−2.575	0.010
Likes	Perceived severity	61.00	112.00	74.914.000	−4.356	<0.001
Comments	Perceived severity	6.00	10.00	75,207.500	−4.291	<0.001

*Test statistic for Mann-Whitney U test.

Perceived barriers to Zika preventive behaviors were barely present (2.8%) as a percentage of the total sample and present in just >10% of the posts specifically mentioning Zika preventive measures. Using mosquito repellent was mentioned most frequently, more so than other available options, such as postponing travel to areas with local Zika activity or wearing long-sleeved shirts or pants. This finding makes sense because half of all preventive measure posts originate with commercial accounts, which often are promoting mosquito repellents. In addition, using mosquito repellent is not a complex behavior, and few barriers to its use likely exist beyond mild inconvenience. However, when a Zika vaccine becomes available, the conversation about Zika preventive measures on Instagram will likely change because vaccination is not without controversy. Finally, cues to action were present in only 10.2% of the sample, and cues to self-efficacy were present in only 9.6% of the sample. Public health communications professionals should focus on increasing these forms of messaging on social media, especially when a vaccine becomes available.

Overall, the Zika-focused posts in this sample reflected a high level of perceived threat and a low level of expressed self-efficacy. At least some of the responses seem to be maladaptive in nature. To counter this trend, public health organizations should consider increasing their activity regarding Zika prevention on Instagram. For example, they could emphasize the benefits and relative ease of restricting travel to high-risk areas, using repellent, and wearing protective clothing—and that the benefits of such actions outweigh the barriers. Because the salience of Zika tends to wane after the summer, cues to action are particularly needed to remind the public of ongoing risk, especially travel-related risk. Last, once a vaccine becomes available, it will be essential to promote the safety and efficacy of the vaccine and counter misinformation about vaccination side effects more generally.

Appendix Additional information on using the Health Belief Model to analyze Instagram posts about ZIka for public health communications.
