# Supplementary material for: Using the Health Belief Model to Analyze Instagram Posts about Zika for Public Health Communications
Source: Emerg Infect Dis. 2019 Jan;25(1):179–80. doi: 10.3201/eid2501.180824 (PMC6302587; doi:10.3201/eid2501.180824)
Supplement: Appendix — Additional information on using the Health Belief Model to analyze Instagram posts about ZIka for public health communications. [file 18-0824-Techapp-s1.pdf]

Article DOI: https://doi.org/10.3201/eid2501.180824

Using the Health Belief Model to Analyze Instagram Posts about Zika for Public Health Communications

Appendix

Health Belief Model

Health behavior theories provide a way to better understand health-related behaviors and design effective public health messaging (*1*). The Health Belief Model (HBM) provides a theoretical framework to help explain and predict the uptake of preventative behaviors. The HBM posits that persons will engage in a health-protective action if they 1) believe that a negative health condition can be avoided; 2) believe that, by taking a recommended action, they will avoid a negative health condition; and 3) believe that they can successfully take a recommended health action. According to the model, a person’s likelihood of engaging in health-promoting behavior (or reducing health risk behavior) is determined by 4 main antecedents: 1) perceived susceptibility to a certain health condition; 2) perceived severity of the health condition and its consequences; 3) perceived barriers to engaging in the advised action; and 4) perceived benefits to taking health action. Together, these 4 perceptions are theorized to account for individuals’ readiness to take health-related action, and are activated by 1) cues to action and 2) self-efficacy to successfully perform the action (*2*). There is strong empirical support for the use of HBM as a framework for developing health education and promotion messages and campaigns (*1*). However, to our knowledge, the model has not yet been applied to Zika-specific health behaviors. Although 2 earlier content analysis involving Zika-related posts on Instagram have been published (*3*,*4*), neither included the HBM or other health behavior theories and theoretical constructs.

Research Questions

To better understand how to communicate with the public about the Zika virus and about Zika preventative measures on Instagram using HBM constructs, it is necessary to identify how persons respond to existing Zika messages on the platform. Given the current growth of Instagram as a platform for public discussions and sharing of information, exploring users' engagement with HBM constructs as part of Zika-related content can yield useful insights for the development of timely and relevant health messages. Therefore, our 2 research questions are 1) What HBM constructs are present in Zika-focused posts on Instagram, and 2) How do users engage with posts containing HBM constructs?

Methods

We analyzed Zika-related posts on the social media platform Instagram by using quantitative content analysis. During August 1–31, 2016, we collected Instagram posts by using #Zika and #ZikaVirus hashtags by using a web-based social media mining tool (https://netlytic.org/), which uses the Instagram Application Programming Interface search/posts endpoint and returns a collection of Instagram posts matching a specified query. Netlytic retrieved the first unique 100 Instagram posts for #Zika and the first unique 100 Instagram posts for #zikavirus every hour, 24 hours a day, for 31 days. At the end of this collection, simple random sampling was used to collect 1,000 distinct Instagram posts from the larger sample of 100,000. The results of the selected hashtags were imported into an Excel (Microsoft, https://www.microsoft.com/en-us) spreadsheet. The unit of analysis was the complete Instagram post: the visual and the accompanying caption.

Coding protocols for the content analysis were developed, tested, and implemented for the coding process. Posts were coded for Instagram-specific variables such as hashtags, mentions, and visual type; Instagram engagement variables in the form of likes and comments; Web site connection; HBM variables (perceived benefits, perceived barriers, perceived severity, perceived susceptibility, self-efficacy, and cues to action); and for the language of the post. The main coder is fluent in English, Dutch, and German and has working knowledge of French and Spanish. Posts in French, Spanish, and Portuguese were translated by a native speaker before being coded. No other languages were present in the study sample.

Applied to Zika and Zika-preventative behaviors (because no treatment except for supportive care is currently known for Zika), the HBM constructs are operationalized as follows: perceived benefits (Appendix Table 1) of the Zika preventative measures, such as protection against disease; perceived barriers (Appendix Table 2) to Zika preventative measures, such as complex instructions and the presence of conspiracy theo ries; perceived susceptibility to the Zika virus; perceived severity of Zika (Appendix Table 3) including expressed fear of Zika; self-efficacy related to Zika preventative measures; and cues to action related to Zika preventative measures, such as a physician's recommendation or an advertisement encouraging the use of mosquito repellent.

Two coders were trained and coded 10% of 100 posts for intercoder reliability. After pretesting and subsequent changes to the coding protocol, the intercoder reliability test with the ReCal statistical program (http://dfreelon.org/utils/recalfront/) showed an average Scott (*5*) π value of 0.82. The individual coefficients were all considered to be reliable, and the lowest coefficient was 0.71 (a complete list is available from the authors). Once reliability was reached, the first coder coded the remaining 900 posts for all study variables.

Statistical Analyses

We used Mann-Whitney U tests to check for differences in Instagram engagement between posts with versus without a range of dichotomous variables. We used Kruskal-Wallis tests to check for differences in Instagram engagement between posts with versus without a range of nominal variables. For both tests, distributions of the engagement frequencies were evaluated and found similar on the basis of visual inspection of a box plot for all variables involved. Whenever the Kruskal-Wallis tests found statistically significant differences, we performed post-hoc analyses by using the procedure of Dunn (*6*) with a Bonferroni correction for multiple comparisons. We present adjusted p values.

Results

Most (75.0%, 750) posts were in English. An additional 18.7% (187) were in Portuguese, 3.7% (37) in Spanish, 1.2% (12) in other languages, and 1.4% (14) consisted of 2 languages. Almost half (49.3%, 493) of the posts were published on individual Instagram accounts, 30.7% (307) originated with commercial/organizational accounts, 8.9% (89) were published by public

health Instagram accounts, 7.5% (75) by news organization accounts, and 1.1% (11) by users identified as medical professionals, (i.e., physicians and nurses). The remaining 2.5% (25) were published by other types of entities. Finally, 5.7% (57) of posts contained a hyperlink. Among these 57 posts, 35.1% (20), pointed to a commercial Web site, 31.6% (18) to a health-related Web site, and 19.3% (11) to a news Web site.

A total of 37.1% (371) posts mentioned mosquitos, 10.0% (100) mentioned microcephaly, and 2.4% (24) mentioned Zika symptoms. Although 7.4% (74) portrayed or mentioned a current Zika patient, only 0.5% (5) of the Instagram posts mentioned a future Zika vaccine and the fact that it was not (yet) available. Finally, 74.7% (747) of posts indicated fear of the Zika virus (Appendix Figure 1). Preventative measures related to Zika were present in 23.8% (238) of the sample. The mean number of likes for this sample was 2,830.1 (SD = 15,178.84, R = 253,000). Because these numbers clearly indicate outliers among the number of likes, we also report the median value of 72.0. The mean number of comments was 65.0 (SD = 212.79, R = 1,938); again, these numbers indicate the presence of outliers. The median for the number of comments was 6.0.

Research Question 1

The first research question asked what HBM constructs were present in Zika-focused posts on Instagram. We report results by HBM construct.

Perceived Severity

The following variables were used to determine perceived severity of the Zika virus: fear of the Zika virus, getting sick because of Zika, serious complications of Zika for a pregnant woman and her fetus, fear of getting pregnant because of Zika, mention of microcephaly, visual presentations of microcephaly, mention of Zika being deadly, and mention of risk to pregnant women (for this and the other HBM constructs, posts could be in >1 category). We show the presence of each of these variables in the Instagram posts of this sample (Appendix Figure 2). A total of 75.8% (758) of Instagram posts in this sample mentioned the perceived severity of Zika.

Perceived Susceptibility

The following variables were used to measure perceived susceptibility (Appendix Table 4) to the Zika virus: high chance of contracting Zika when in an area with a lot of mosquitoes,

living in an area with ongoing local Zika transmission, traveling to an area with ongoing local Zika transmission, living in an area with a lot of mosquitoes, mention of travel restrictions, mention of sexual transmission (Appendix Figure 3), and mention that getting infected with Zika is currently a possibility. We show the presence of each of these variables in the Instagram posts of this sample (Appendix Figure 4). A total of 59.9% (599) of Instagram posts in this sample mentioned the perceived susceptibility of Zika virus.

Perceived Benefits

The following variables were used to measure perceived benefits of Zika preventative measures: benefits of use of mosquito repellent (Appendix Figure 2), benefits of postponing travel to Zika-infected areas, benefits of avoiding travel to Zika-infected areas, benefits of wearing long sleeves and long pants when outdoors, and benefits of using condoms. We determined the presence of each of these variables in the Instagram posts of this sample (Appendix Table 1). In total, 21.7% (217) of Instagram posts in this sample mentioned the perceived benefits of Zika preventative measures. However, of the number of posts that mentioned Zika preventative measures in general, 91.2% (217) mentioned perceived benefits of these measures.

Perceived Barriers

The following variables were used to gauge perceived barriers to Zika prevention: lack of funds for Zika prevention, complex instructions for Zika prevention, questioning the safety of Zika preventative measures, Zika preventions being unrealistic, Zika being hard to prevent, and conspiracy theories related to Zika. We determined the presence of each of these variables in the Instagram posts of this sample (Appendix Table 2). Of particular interest is that ≈10% of the sample discussed conspiracy theories related to the Zika virus (Appendix Figure 4).

Cues to Action and Self-Efficacy

Mentions of cues to action (Appendix Figure 1) for Zika preventative measures were present in 10.2% (102) of the posts. Mentions of self-efficacy related to Zika preventative measures were present in 9.6% (96) of the Instagram posts.

Research Question 2

The second research question asked how users engaged with Zika-related Instagram posts that contain HBM constructs. We performed Mann-Whitney U tests to determine whether there were differences in likes and comments between posts with the presence versus absence of HBM constructs (i.e., perceived benefits, perceived barriers, perceived susceptibility, perceived severity, perceived self-efficacy, and cues to action). Like ($p = 0.023$) and comment ($p = 0.010$) frequencies were significantly lower in posts that mentioned perceived benefits of Zika prevention and that mentioned the perceived severity of Zika versus posts that did not ($p<0.001$ for both likes and comments). In addition, like ($p<0.001$) and comment ($p<0.001$) frequencies were significantly higher in posts that mentioned conspiracy theories related to the Zika virus than in posts that did not. There were no major differences between Instagram engagement metrics for perceived susceptibility, self-efficacy, and cues to action (Appendix Table 3).

References

1. Glanz K, Rimer BK, Lewis ML. Health behavior and health education: theory, research, and practice. San Francisco: Jossey-Bass; 2015.

2. Janz NK, Becker MH. The Health Belief Model: a decade later. Health Educ Q. 1984;11:1–47. PubMed http://dx.doi.org/10.1177/109019818401100101

3. Fung IC-H, Blankenship EB, Goff ME, Mullican LA, Chan KC, Saroha N, et al. Zika-virus-related photo sharing on Pinterest and Instagram. Disaster Med Public Health Prep. 2017;11:656–9. PubMed http://dx.doi.org/10.1017/dmp.2017.23

4. Seltzer EK, Horst-Martz E, Lu M, Merchant RM. Public sentiment and discourse about Zika virus on Instagram. Public Health. 2017;150:170–5. PubMed http://dx.doi.org/10.1016/j.puhe.2017.07.015

5. Scott WA. Reliability of content analysis: the case of nominal scale codin*g*. Public Opinion Quarterly. 1955;19:321–5. http://dx.doi.org/10.1086/266577

6. Dunn OJ. Multiple comparisons using rank sums. Technometrics. 1964;6:241–52. http://dx.doi.org/10.1080/00401706.1964.10490181

Appendix Table 1. Perceived benefits of Zika preventative measures

Variable	No. (%)
Use of mosquito repellent	157 (15.7)
Postponing travel to areas with Zika	19 (1.9)
Avoiding travel to areas with Zika	44 (4.4)
Wearing long sleeves and long pants when outdoors	33 (3.3)
Using condoms	12 (1.2)

Appendix Table 2. Perceived barriers to Zika preventative measures

Variable	No. (%)
Lack of funds for Zika prevention	7 (0.7)
Complex instructions for Zika prevention	3 (0.3)
Questioning the safety of Zika preventative measures	10 (1.0)
Zika preventions are unrealistic	3 (0.3)
Zika is hard to prevent	7 (0.7)
Conspiracy theory	95 (9.5)
Governmental conspiracy	93 (9.3)
Pharmaceutical conspiracy	63 (6.3)
Medical conspiracy	42 (4.2)

Appendix Table 3. Perceived severity of Zika

Variable	No. (%)
Fear of Zika virus	747 (74.7
Getting sick because of Zika	29 (2.9)
Serious Zika complications for a pregnant woman and her fetus	115 (11.5)
Fear of getting pregnant because of Zika	12 (1.2)
Mention microcephaly	100 (10.0)
Microencephaly visual	44 (4.4)
Zika is deadly	4 (0.4)
Mention of risk to pregnant women	126 (12.6)

Appendix Table 4. Perceived susceptibility to Zika

Variable	No. (%)
High chance of contracting Zika when in an area with a lot of mosquitoes	107 (10.7)
Living in area with local Zika transmission	327 (32.7)
Traveling to area with local Zika transmission	160 (16.0)
Mention of travel restrictions	31 (3.1)
Mention of sexual transmission	23 (2.3)
Living in area with a lot of mosquitoes	248 (24.8)
Zika infection is currently a possibility	352 (35.2)

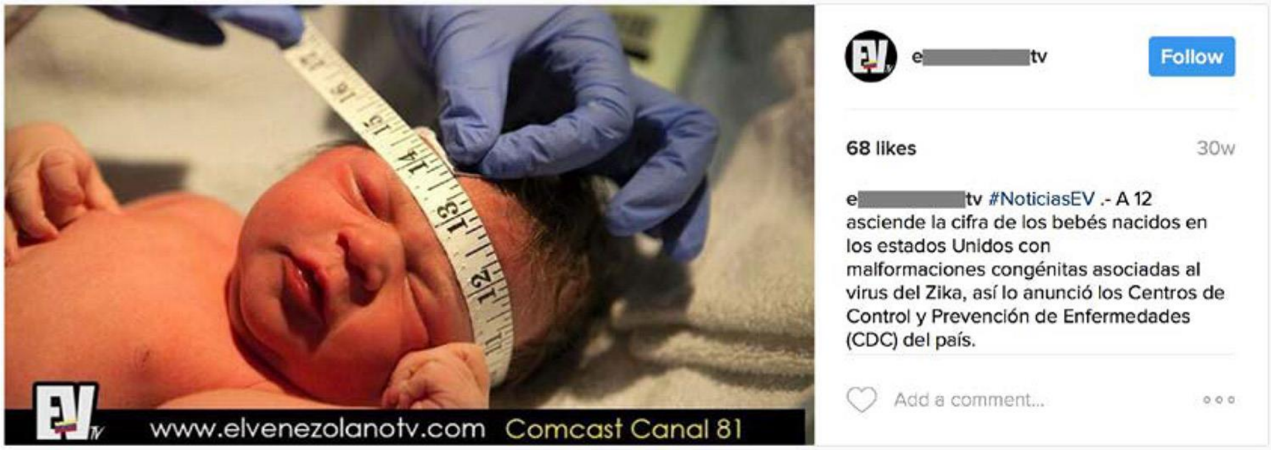

Appendix Figure 1. Example of fear of Zika.

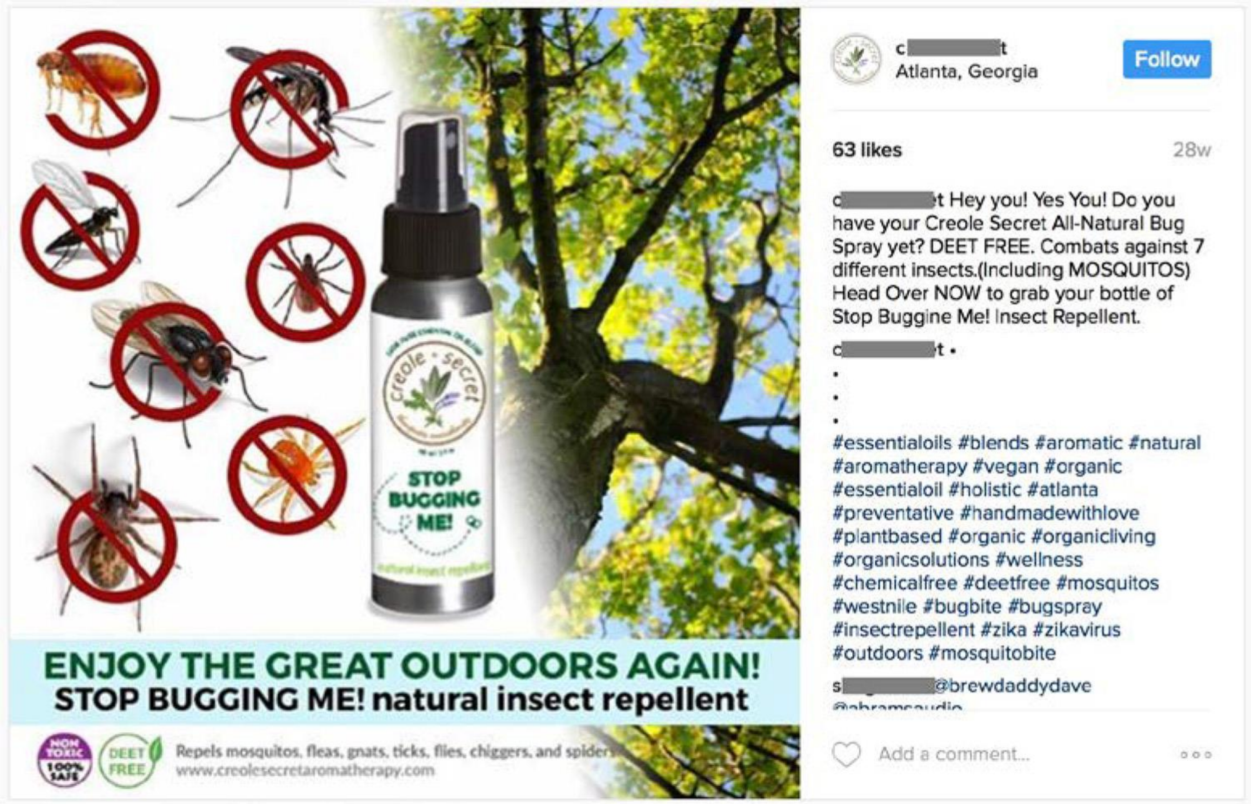

Appendix Figure 2. Example of perceived benefits/cues to action for Zika.

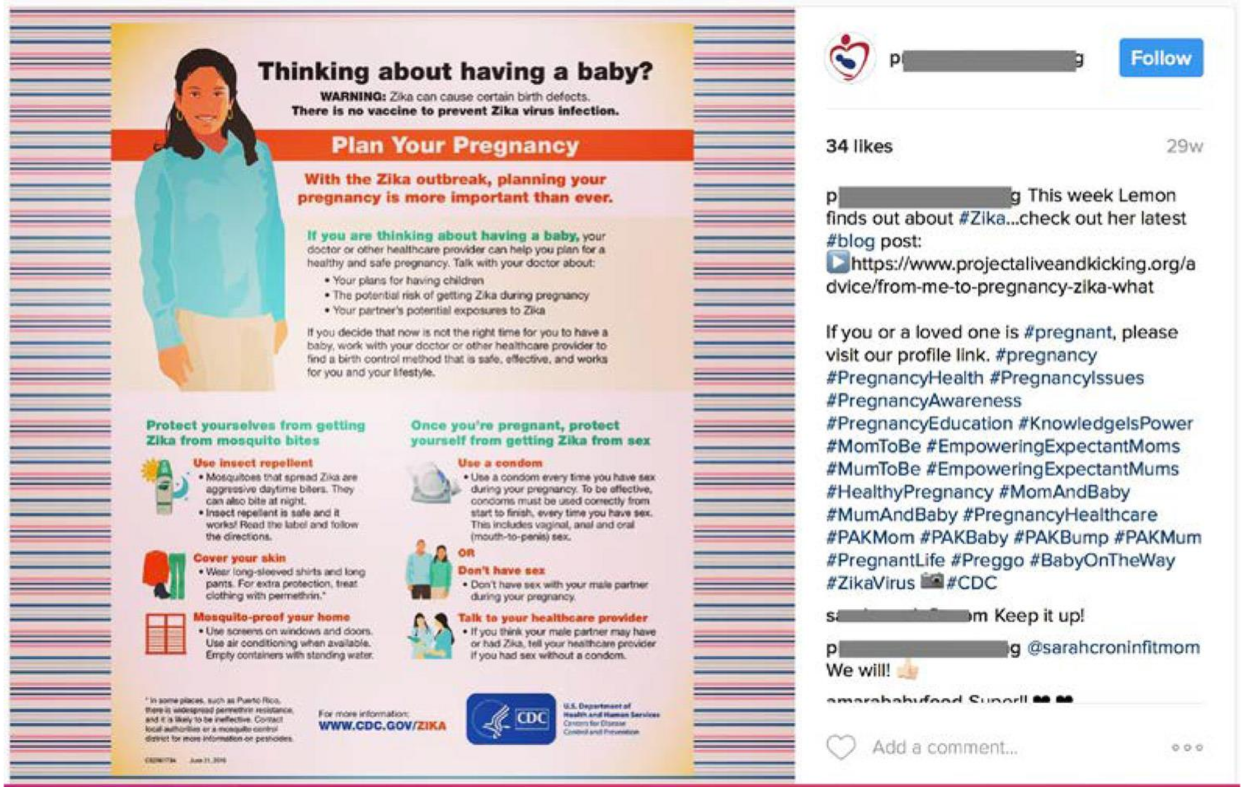

Appendix Figure 3. Example of sexual transmission of Zika.

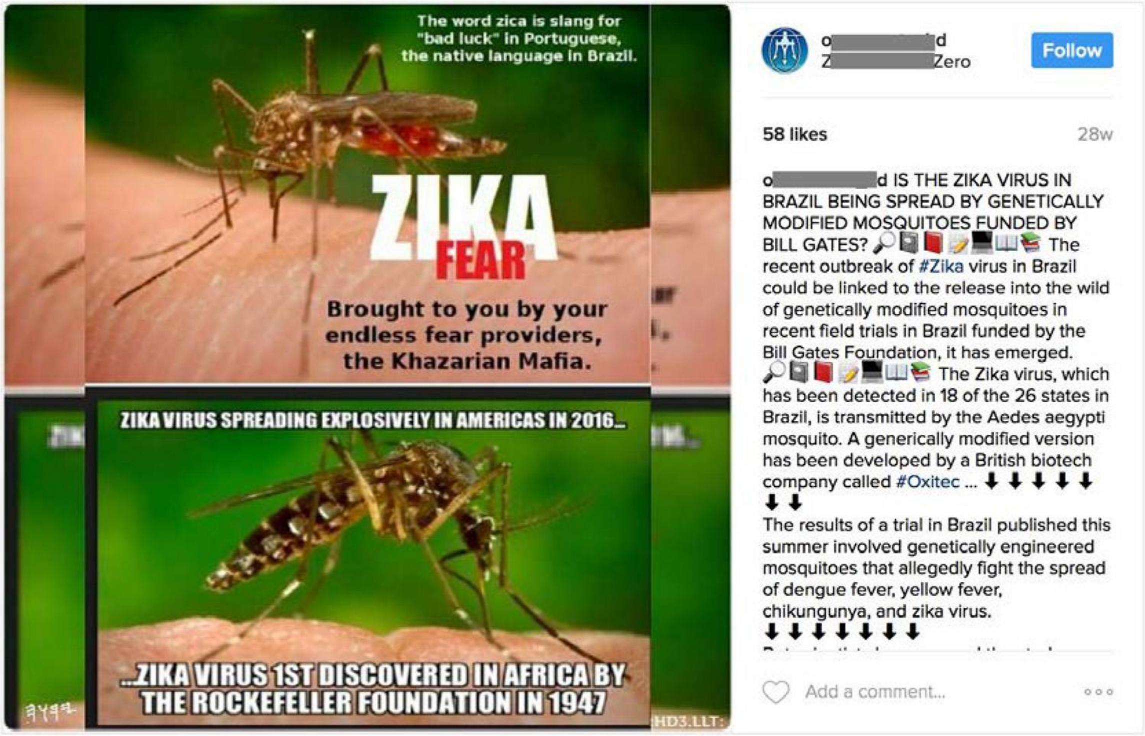

Appendix Figure 4. Example of government conspiracy regarding Zika.
